# Challenges for the surgical capacity building of township hospitals among the Central China: a retrospective study

**DOI:** 10.1186/s12939-018-0766-4

**Published:** 2018-05-02

**Authors:** Zhong Li, Jian Yang, Yue Wu, Zijin Pan, Xiaoqun He, Boyang Li, Liang Zhang

**Affiliations:** 1 School of Medicine and Health Management, Tongji Medical College, Huazhong University of Science and Technology, No 13 Hangkong Road, Qiaokou District Wuhan, Hubei, 430030 China; 2 Department of Medical Affairs, Guangdong General Hospital, Guangzhou, 510080 Guangdong China

**Keywords:** Township hospital, Surgical capacity, Pay for performance, Integrated delivery networks, Rural China

## Abstract

**Background:**

China’s rapid transition in healthcare service system has posed considerable challenges for the primary care system. Little is known regarding the capacity of township hospitals (THs) to deliver surgical care in rural China with over 600 million lives. We aimed to ascertain its current performance, barriers, and summary lessons for its re-building in central China.

**Methods:**

This study was conducted in four counties from two provinces in central China. The New Rural Cooperative Medical System (NRCMS) claim data from two counties in Hubei province was analyzed to describe the current situation of surgical care provision. Based on previous studies, self-administered questionnaire was established to collect key indicators from 60 THs from 2011 to 2015, and social and economic statuses of the sampling townships were collected from the local statistical yearbook. Semi-structured interviews were conducted among seven key administrators in the THs that did not provide appendectomy care in 2015. Determinants of appendectomy care provision were examined using a negative binominal regression model.

**Results:**

First, with the rapid increase in inpatient services provided by the THs, their proportion of surgical service provision has been nibbled by out-of-county facilities. Second, although DY achieved a stable performance, the total amount of appendectomy provided by the 60 THs decreased to 589 in 2015 from 1389 in 2011. Moreover, their proportion reduced to 26.77% in 2015 from 41.84% in 2012. Third, an increasing number of THs did not provide appendectomy in 2015, with the shortage of anesthesiologists and equipment as the most mentioned reasons (46.43%). Estimation results from the negative binomial model indicated that the annual average per capita disposable income and tightly integrated delivery networks (IDNs) negatively affected the amount of appendectomy provided by THs. By contrast, the probability of appendectomy provision by THs was increased by performance-related payment (PRP). Out-of-pocket (OOP) cost gap of appendectomy services between the two different levels of facilities, payment method, and the size of THs presented no observable improvement to the likelihood of appendectomy care in THs.

**Conclusion:**

The county-level health system did not effectively respond to the continuously increasing surgical care need. The surgical capacity of THs declined with the surgical patterns’ simplistic and quantity reduction. Deficits and critical challenges for surgical capacity building in central China were identified, including shortage of human resources and medical equipment and increasing income. Moreover, tight IDNs do not temporarily achieve capacity building. Therefore, the reimbursement rate should be further ranged, and physicians should be incentivized appropriately. The administrators, policy makers, and medical staff of THs should be aware of these findings owing to the potential benefits for the capacity building of the rural healthcare system.

## Background

Injury caused numerous disability and mortality cases globally, especially in conflict-affected or resource-limited regions [1, 2]. Approximately 11% of the total disability-adjusted life year worldwide could be attributed to the surgical disease burden [3]. As a significant part of global health [4, 5], evidence proved that essential surgical care provision could effectively satisfy the demand of local residents [6–10] and benefit universal health coverage [11, 12]. However, many low-and middle-income countries (LMICs) could still not provide essential surgical services, mainly caused by limited health human resources, logistics, medicine, infrastructure, equipment, and supplies [5, 13–16]. Such restriction further prevented two billion people from gaining access to surgical and anesthesia care [1, 14, 17]. The accessibility of surgical care varied markedly worldwide [12, 18, 19].

Although no standard existed for the required capabilities of the first-level hospitals, the caesarean delivery, laparotomy, and open fractures are widely recognized as the bellwether procedures of their essential surgical care [18, 20]. In addition, medical staffs were trained to provide these services worldwide [10, 21, 22]. In 2004, WHO launched the Emergency and Essential Surgical Care Project to strengthen the availability of essential surgical care, including burns, orthopedics, obstetrical acute conditions, and lower abdominal diseases [4, 23–25]. This project achieved huge success on the surgical care provision in the primary care system and proved cost- effective [12, 26].

After the establishment of New China, the three-tiered health delivery network was quickly formed, led by the county-level hospitals (CLHs) and composed of THs and village clinics [27]. Different institutions coordinated with one another and provided healthcare services together. The village clinics, as the cornerstone of the network, provide essential services to enrollers through the Cooperative Medical System. THs and CLHs are responsible for the continuing education and technical assistance for the medical staff in THs. With the huge changes in both population patterns and disease spectra, the following decades have witnessed its significant achievements [28–33]. However, from the Reform and Opening-up, the two-referral services were heavily damaged and residents freely jumped to high level facilities for healthcare services. Different institutions are pursuing their own interests. A poorly equipped referral system, such as other LMICs, caused many problems [17]. Issues included surgical services neglected in the primary care facilities, which are necessary to encourage the provision of related services by the primary care system [34]. In addition, the dysfunction of THs caused an increasingly pressing fragmented three-tiered healthcare delivery system [35]. First, secondary and tertiary hospitals have excessively provided services outside its functional orientation under the side effects of a market-oriented system [36]. Second, the patients’ distrust on primary care facilities has also caused its poor performance [37]. According to the fifth National Health Services Survey and China Health Statistic Yearbook, the proportion of hospitalization in THs decreased to 29.8% in 2013 from 36.6% in 2008, whereas the proportion of the CLHs increased from 50.0% to 55.7%. The beds of THs in the entire health system for the surgeon, gynecology, and obstetrics in THs decreased from 17.58% and 12.81% in 2011 to 15.9% and 10.23% in 2015, respectively [38, 39]. Third, medical staffs in the primary facilities were not fully motivated, attending to be more or less single-minded on the chronic disease management services [40–42]. Moreover, compared with urban areas providing a broader range of surgical services, geographic disparities startlingly exist with respect to access to healthcare services in rural China [43]. Hence, surgical capacity and its availability in the rural areas must be urgently improved, considering there still existing 603 million lives [4, 44].

Since 2009, the New Healthcare Reform has strengthened the capacity building of the grassroots facilities. The amount of 100 billion RMB was placed into county-level and below facilities [45]. With the implementation of separating revenue and expenditure (SRE), pay for performance (P4P), zero mark-up of medicine, specific medical students for the rural areas [46], ranged reimbursement ratio, and IDNs [47], the quality of care and patients’ satisfaction improved [48], but a few of the above policies caused burnout or mobility among the medical staffs [36]. In recent years, approximately 30% of physicians’ salaries, which were previously linked with the title, working years, and education background, were linked with the amount of service provision in certain THs to counteract the egalitarianism caused by the SRE and P4P. To distinguish it from P4P in this study, we reported it as PRP.

Abundant research has highlighted the role of incentives, IDNs in the healthcare reform, especially chronic disease management [48–50]. However, existing research has ignored the role of surgical care in the Chinese rural health system. Moreover, no exact data could present an evolution of surgical care provision or have explored its determinants with the increasing number of surgical care necessary. This study aims to quantify the surgical capacity from the providers’ perspective and the patients’ flow and to identify its deficits and challenges in multiple channels with the appendectomy provision from 2011 to 2015 [34]. Such goal acts as the first step to promote capacity building in the rural healthcare system.

## Methods

### Study sample

**Fig. 1:**
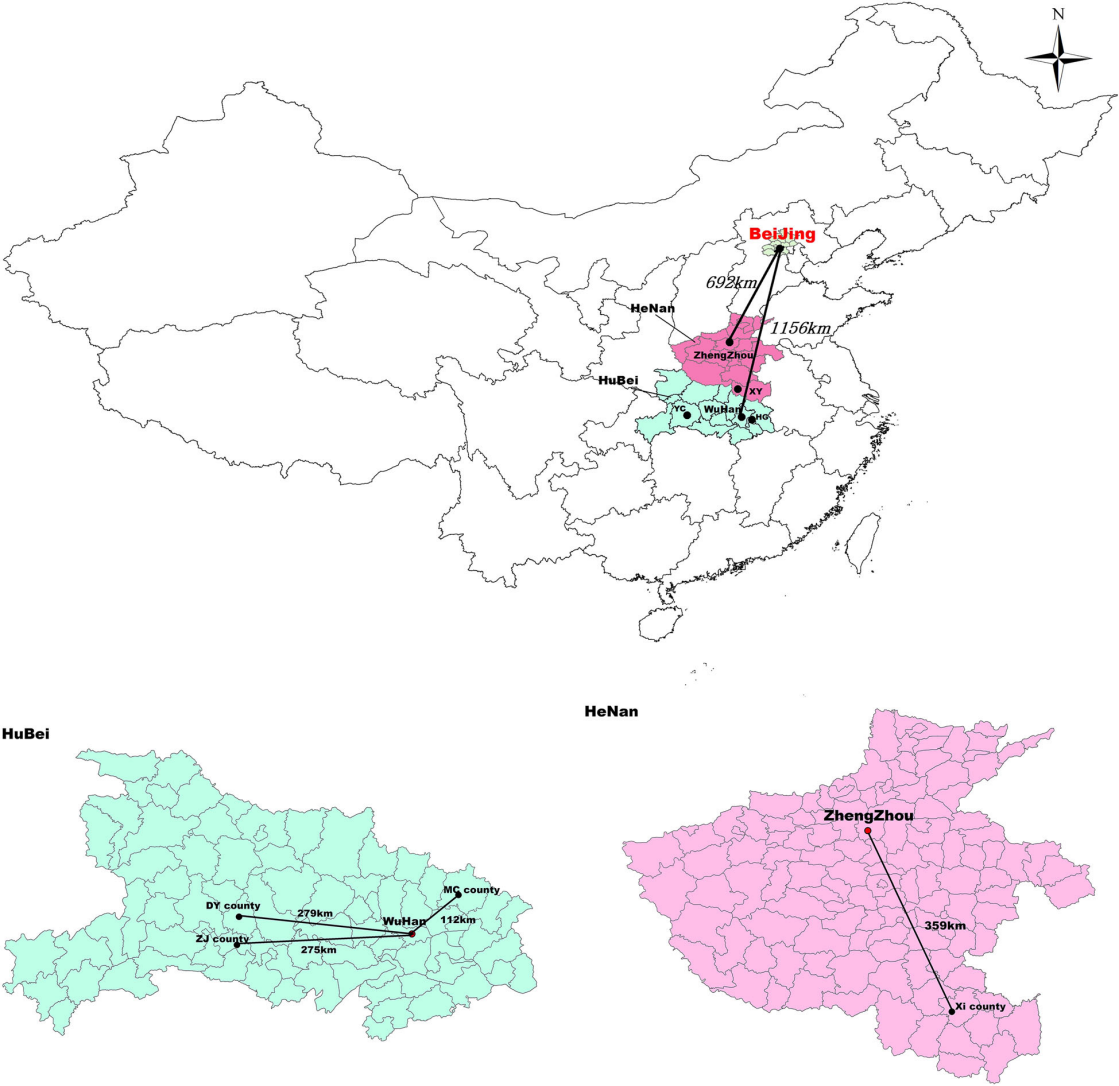
Map of the four counties enrolled

The selection of 60 THs from four counties was conducted based on a multistage stratified purposive sampling method [51–53]. First, the six provinces, namely, Shanxi (23/31), Henan (17/31), Anhui (18/31), Hubei (10/31), Jiangxi (13/31), and Hunan (15/31), were classified into two groups according to the ranking of per capita disposal income in 2015. We then randomly selected two provinces from the two groups. Second, given the huge county-level variations within the province on the socio-economic development, 102 county-level regions in Hubei and 158 county-level regions in Henan were divided into two groups based on the per capita disposal income. Macheng (MC), Zhijiang (ZJ), and Dangyang (DY) in Hubei, and Xi (XI) in Henan were then selected (see Fig. 1 and Table 1). Finally, all 60 THs were selected to finish the self-administrated questionnaire. Seven THs that did not provide the appendectomy care were selected for our semi-structured interviews (see Table 2).

**Table 1:** Social economic status and other characteristic of sampling counties in 2015

Characteristic	XI	MC	DY	ZJ
Income level	Undeveloped	Undeveloped	developed	developed
Number of THs	21	21	10	8
Annual average per capita disposable income (¥)	8516.71	8080.86	15,869.7	15,578.88
Number of outpatients	35,750.43	67,369.1	51,201.4	39,350.13
Number of inpatients	1578.76	2829.86	2195.8	2480.25
Average time to the THs (min)	23	354	33	28
Average time to the local People hospital (min)	46	50	35	49
Average cost of appendicitis per 1000 (¥)	2312.95	1447.99	1954.31	2980.78
Average cost gap between CLHs and THs of appendicitis (¥)	819.62	1233.86	1784.7	1449.88
Average number of anesthesiologist per 1000	0.81	1.52	1.1	1.38
Average number of surgeon per 1000	2.38	2.57	3	2.63

Note: Time to the THs and local People hospital using the car was estimated with the Baidu Map

**Table 2:** Respondents’ characteristic of the self-administrated questionnaires and semi-structured interviews

Characteristic		Respondents of the self-administrated questionnaire (*N* = 60)	Respondents of semi-structured interviews (*N* = 7)
Gender	male	47	6
Age(years)		44.41 ± 4.70	44.56 ± 5.23
Years practicing as physicians		24.36 ± 4.79	23.57 ± 5.48
Education	Undergraduate	27	3
	College and above	33	4

### Data collection procedure

Based on previous studies [54, 55], the questionnaire comprised the following three parts: 1) social and economic statuses of the sampling townships, including total population and annual per capital disposable income; 2) basic information of THs, including the numbers of anesthesiologists and nurses, medicine and anesthetics or surgical facilities and the amount of appendectomy provision; 3) Institutional operation, including the type of IDNs, size of facilities, PRP or not, average OOP cost gap between the township-county hospitals, and payment method. This survey was conducted from August 2016 to October 2016. First, the NRCMS claim data and related policies from 1st January 2011 to 31st December 2015 were retrieved, except from the claim data of MC from 1st January 2011 to 31st December 2011. Second, the socio-economic characterization of the sampling townships was collected from the local statistical yearbooks. The self-developed questionnaire was used to collect basic information and institutional operation the 60 THs from the deputy dean or head of the surgical department. Subsequently, the semi-structured interviews were conducted among the administrators of the seven THs that did not provide appendectomy care by the principal investigator.

### Statistical analysis

First, descriptive analysis was applied in the claim and survey data to visualize the patient flow within the county-level healthcare delivery system. We used the χ^2^ or Cochran-Armitage trend test to determine statistical significance. Second, we summarized the potential determinants of the phenomenon behind why certain THs did not provide appendectomy. Third, considering the over-dispersed appendectomy provision, a negative binomial regression model was used to investigate its determinants [56–58] (see Table 3), which were extensively used in similar studies [59–61]. Cost data were adjusted by the CPI of the 2015 National Health Statistic Yearbook [39]. Data were managed by Epidata 3.0 and analyzed by Stata 13.0 (Stata Corp LP, College Station, TX, USA). *P*-value of < 0.05 was defined as statistical significance.

**Table 3:** Variables description

Variable	Maximum	Minimum	Mean	Std
Noapp	171	0	17	25
size	3	1	1.78	0.63
income	18,526	2267	8447	3486
costgap	3614	447	1177	398
p4p	1	0	0.69	0.46
Payment	3	1	1.8	0.55
IDNs	4	1	1.63	0.93

Note: Noapp, the number of appendicitis provided by the township hospitals annually; Size can represent its inpatient and surgical services’ capacity: street township hospitals = 1, ordinary THs = 2, central THs =3; Costgap, the difference between the average out-of-pocket cost of appendicitis in the THs and CLHs; The remuneration of medical staff are linked up with the healthcare services provision in a certain degree. Payment methods were composited with single-disease with abundant quota, single-disease with limited quota, fee for service. IDNs are vigorously promoted by the China government, the sampling THs were clustered as four types based its property right belongings and management form: no integration = 1, tight integration = 2, loose integration = 3, merged integration = 4

## Results

### Characteristic of surgical care and appendectomy provision

**Fig. 2:**
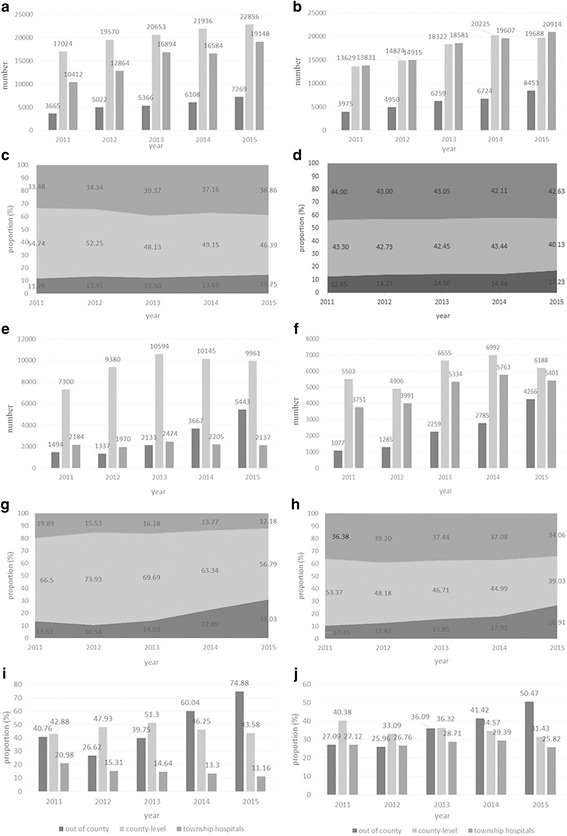
Surgical care provision. Note: (**a** and **b**) Inpatients care within the three-level facilities in DY and ZJ; (**c** and **d**) Inpatients care proportion within the three-level facilities in DY (*P* < 0.001) and ZJ (*P* < 0.001); (**e** and **f**) Surgical care in the three-level facilities in DY and ZJ; (**g**) (**h**) Surgical cares’ proportion among the three-level facilities in DY(*P* < 0.001) and ZJ (*P* < 0.001); (**i** and **j**) Proportion of inpatients care utilized surgical services within the three-level facilities in DY (*P* < 0.001) and ZJ (*P* < 0.001)

Limited to data quality and accessibility, the NRCMS claim data of the DY and ZJ were selected to sketch the patient flow. From 2011 to 2015, a proportion of the inpatient services provided by THs in DY increased from 33.48% to 38.86% (*P* < 0.001), whereas that of ZJ decreased from 44.00% to 42.63% (P < 0.001) (see Fig. 2). The inpatient services provided by out-of-county hospitals grew rapidly, accounting for 14.75% and 17.23% (*P* < 0.001). However, under rapid growth in the surgical service provision among the three-level facilities, the proportion of THs’ provision declined to 12.18% and 34.06% from 19.39% (*P* < 0.001) and 36.38% (*P* < 0.001), respectively. The out-of-county facilities are rapidly nibbling their proportion, accounting for 31.03% (P < 0.001) and 26.91% (P < 0.001) in 2015, respectively. Moreover, the proportion of inpatients utilizing surgical care in the THs decreased to 11.16% (P < 0.001) and 25.82% (P < 0.001). Out-of-county facilities increased dramatically to 31.03% (P < 0.001) and 26.91% (P < 0.001) from 13.61% and 10.45%, respectively.

**Fig. 3:**
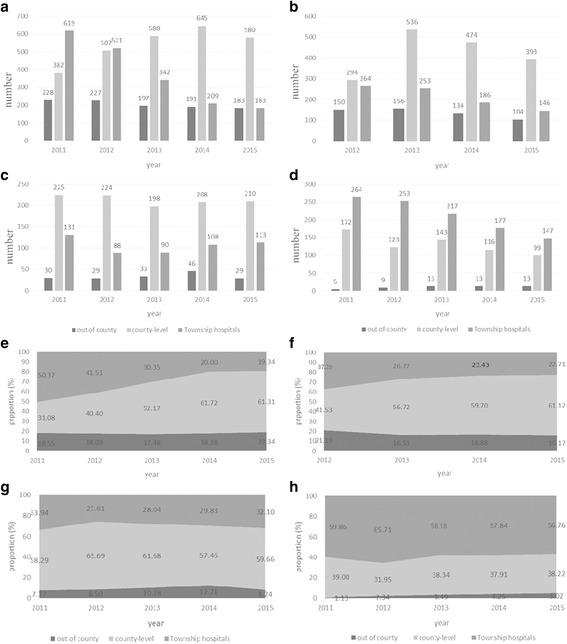
Appendectomy care provision. Note: (**a**-**d**) Appendectomy care within the three-level facilities in XI, MC, DY and ZJ; (**e**-**h**) Appendectomy care proportion within the three-level facilities in XI (P < 0.001), MC (P < 0.001), DY (*P* = 0.862) and ZJ (*P* = 0.007)

The amount of appendectomy provided by the 60 THs has decreased to 589 in 2015 from 1389 in 2011 (see Fig. 3). Appendectomy of XI (P < 0.001), MC (P < 0.001), and ZJ (*P* = 0.007) decreased from 619, 264, and 264 to 183, 146, and 147 cases, respectively. By contrast, DY’s performance was stable (*P* = 0.862). The number of THs that cannot provide appendectomy increased to 7 (11.67%) in 2015 from 5 (8.33%) in 2011 (*P* = 0.5428). The number of THs, which does not provide appendectomy even if equipped with related resources increased to 9 (15.00%) in 2015 from 1(1.67%) in 2011 (*P* = 0.0082).

### Determinants of the appendectomy care provision

In 2015, seven THs cannot provide appendectomy services. Table 4 summarizes the most frequently emerging reasons. Shortage of anesthesiologists and equipment is apparently the most cited reasons, accounting for 46.43%. The negative binominal regression model was conducted to explore determinants of appendectomy counts (see Table 3). According to the results of Hausman test (chi2 [8] =5.00, Prob > chi2 = 0.757), we choose the random effect model [62]. As Table 5, the annual average per capita disposable income (RR = 0.892, *P* < 0.001) negatively affected the TH’s appendectomy provision. Effects of the OOP cost gap between the two different levels of facilities (RR = 1.000, *P* = 0.671) and payment method (RR_SDLQ_ = 0.871, *P* = 0.146, RR_FFS_ = 0.952, *P* = 0.816) are statistically insignificant. Moreover, the size of THs did not improve the likelihood of appendectomy provision (RR_ordinary_ = 0.527, *P* = 0.170, RR_central_ = 0.664, *P* = 0.401). The tight IDNs (RR = 0.444, *P* = 0.006) negatively affected the appendectomy provision. By contrast, the PRP has significantly increased THs’ probability of providing appendectomy care relative to the P4P with egalitarianism (RR = 2.206, P = 0.006).

**Table 4:** Self-reported reasons of the 7 township hospitals administrators

Reasons	Frequency	Percent	Accumulative %
Shortage of anesthesiologist	7	25.00	25.00
Shortage of Medical apparatus and instruments	6	21.43	46.43
Shortage of surgeon	4	14.29	60.72
Shortage of nurse	4	14.29	75.01
Shortage of surgical facilities	3	10.71	85.72
Shortage of pharmaceutical capacity	3	10.71	96.43
Shortage of Inspection equipment	1	3.57	100.00

**Table 5:** Results of the negative binomial regression model

Independent variable		Random effect model	
		IRR (95% CI)	*P*
Income		0.897 (0.853, 0.943)	< 0.001
Costgap		1.000 (1.000, 1.000)	0.671
Size	street THs#		
	ordinary THs	0.527 (0.211, 1.316)	0.170
	central THs	0.664 (0.256, 1.727)	0.401
IDNs	No#		
	Tight	0.484 (0.290, 0.810)	0.006
	Loose	1.011 (0.672, 1.522)	0.956
	Merged	0.881 (0.319, 2.433)	0.807
PRP	No#		
	Yes	2.305 (1.570, 3.384)	< 0.001
Payment	SDAQ#		
	SDLQ	0.868 (0.731, 1.031)	0.106
	FFS	0.939 (0.638, 1.382)	0.749
Constant		23.364 (7.920, 68.921)	
Log likelihood		− 875.77356	
Wald chi2(9)		82.57	
Prob>chi2		< 0.001	

Note: *IRR* incidence rate ratio, *SDAQ* single-disease with abundant quota, *SDLQ* single-disease with limited quota, *#*, reference group. According to the Hausman test (chi2(8) =5.00, Prob>chi2 = 0.757), we use the random effect model

## Discussion

This study presents the dramatic decline in the quantity and proportion of surgical services by THs, as county-level surgical service provision continues to rise from 2011 to 2015. The importance of THs is gnawing away, and the expansion of CLHs occupied THs’ space as the THs’ main source of revenue changes from medical service provision to public health services [63]. Such situation is similar to the phenomenon of the largely missing surgical services from the early Medicare Accountable Care Organization in the USA [64]. This gap may be associated with the status of the medical staff in the THs, who seem “fuzzy” on their roles [40]. Moreover, an increasing number of THs are unwilling to provide surgical services, in line with previous studies in which ignorance of medical service provisions is caused by an overemphasis on the chronic disease management in China [47, 48]. The geographic disparity is also impressive. Given that the DY and ZJ counties had once been commended for its tiered healthcare delivery system by the State of Council, we could speculate that a few undeveloped counties may face other austere challenges, and the current situation would also negatively affect the medical quality [65]. In XI county, appendectomy care was included in the coordination care list to improve quality of care [36]. However, the practice did not promote surgical task sharing, which might be associated with the malposition of actual capacity. Extra support should be added on the clinical collaboration between the medical staff and may even require a long period to resolve the shortage, thereby enabling team-based capacity building [66]. A distorted distribution of health utilization in the CLHs resulted in considerable inefficiencies in terms of resource allocation. Overall, practitioners must urgently be well trained and retained to provide surgical services. Moreover, the essential medicine list should be progressively expanded [67].

When appendectomy was taken as an example to explore potential determinants, the panel data analysis demonstrates that appendectomy care provision by the THs was reduced with the increase in per capita disposable income. The OOP cost gap between THs and CLHs could not change the patients’ behavior, in line with one study conducted in rural Gansu Province [68]. Similarly, this condition could also be explained by the awakening health literacy with increasing income [69]. Such scenario may aggravate the growth of patients’ OOP cost and local health spending and expand the disparities with respect to the benefits of health insurance between the poor and the rich [70]. As the main source of different levels of institutions [63], current payment methods failed to fully incentivize physicians in THs to provide appendectomy care, the higher quota reimbursement and FFS cannot encourage physicians to provide further services, reflecting the irrational incentives in the current insurance system. Moreover, with the promising scenario, different kinds of IDNs were promoted across the county to facilitate the cooperation of different institutions with shared responsibility and benefits [71]. However, the tight IDNs reduced surgical care provision, indicating that the coordination between CLHs and THs did not perform as expected. It reminds us that the CLHs must be strictly regulated and that addressing the urgent need of the THs must be a priority during the establishment of IDNs. Additionally, positive effect of PRP on the appendectomy care provision indicates that improve the economic incentive of physicians would attract other physicians to provide surgical services. Hence, the economic leverage should be substantially developed to reduce burnout [72]. Considering the “ambivalent” compensation system for medical staff in China, in remote or rural areas with poor capacity and for specific population, the medical staff should be precisely targeted for empowerment [45, 73].

## Conclusion

To the best of our knowledge, as the first research that attempted to ascertain challenges for surgical care provision in rural China, this insightful study is significant to researchers, policy makers, and THs’ administrators. The current study offers helpful suggestions to facilitate the recovery of the capacity of the rural health system. Surgical services are under unprecedented challenges with a deteriorating trend. The surgical capacity is also under decline because of the inadequate motivation in both salaries and promotion and system deficiency. Considering that healthcare supply has been lagged by the dynamic demand and the payment methods for physicians are hysteretic to the supply, current and following policies should begin from the autonomous capacity building. Moreover, retention and recruitment of medical staff must be considered as priorities.

### Limitations

Given that the first part of our study is limited to quality and accessibility of claim data, we only analyzed data from the DY and ZJ to describe trends in the surgical care provision. Further exploration should concentrate on the regular assessment of the transition base on large amounts of THs, as well as targeted interventions and capacity building.
